# Immediate pre-operative computed tomography for surgical planning of equine fracture repair: A retrospective review of 55 cases

**DOI:** 10.1371/journal.pone.0278748

**Published:** 2022-12-28

**Authors:** Camilla J. Taylor, Vanessa G. Peter, Matthew O. D. Coleridge, Andrew P. Bathe

**Affiliations:** Rossdales Equine Hospital, Newmarket, England, United Kingdom; Cairo University Faculty of Veterinary Medicine, EGYPT

## Abstract

**Introduction:**

Fracture configuration is often more complex than is radiographically appreciable. The objective of this study is to describe the influence of pre-operative computed tomography (CT) for surgical planning in a variety of fracture types. This has not been described in previous studies.

**Materials and methods:**

All cases with pre-operative radiographs, admitted for CT and surgical repair of a suspected limb fracture from January 2010-December 2020 were reviewed. CT was acquired under general anaesthesia in a multi-slice helical scanner; any surgery was then performed immediately. Three diplomates (two surgical; one diagnostic imaging) performed a blinded retrospective review of the radiographs and CT for each horse. A consensus decision was made on any change in surgical plan prior to and after CT review, and cases divided into three categories: CT of major, intermediate or minor relevance, as previously described by Genton *et al*, 2019.

**Results:**

55 cases were collated. Thoroughbred racehorses predominated. The median age was 3 years. A diverse range of fractures were presented: proximal phalanx (18/55), carpal (17/55), metacarpal/tarsal (11/55), sesamoid (5/55), tarsal (3/55), and middle phalanx (1/55). In 13 of 55 cases (23.6%, 95% CI[12%,35%]) CT was of major relevance. In 21 of 55 cases CT was of intermediate relevance (38.2%, 95% CI[25%,51%]). In 21 of 55 cases CT was of minor relevance (38.2%, 95% CI[25%,51%]). A Fisher’s exact test demonstrated no statistical difference in CT relevance between fracture types (p<0.05).

**Discussion/Conclusions:**

This study demonstrates that CT has a significant role in surgical planning, and in the majority (61.8%) of cases added additional information or significantly changed the surgical plan. In all cases CT ensured confidence in surgical planning.

## Introduction

Fractures of the distal limb are relatively common in the equine athlete and poor fracture healing can lead to persistent lameness which often leads to significant economic, welfare or functional losses [1]. Surgical repair of limb fractures aims to maximise return to function by minimising complications relating to fracture healing such as excessive callus formation, osteoarthritis or persistent lameness [2]. In order to achieve this, implant placement must be accurate and well planned to ensure complete reduction of the fracture. In first instance, suspected fractures are assessed radiographically. However, fracture configuration is often so complex, that radiology alone does not provide sufficient clarity. This poses challenges relating to accuracy of surgical planning following radiographic diagnosis [3]. Advanced imaging produces multiplanar images to overcome anatomical superimposition allowing more detailed investigation and clarification of fracture configuration. The use of which, is becoming commonplace to facilitate more accurate surgical planning and provide a more clear prognosis for outcome [4].

The comparative influence of pre-operative CT vs radiology alone, on surgical planning for fracture repair in a variety of fracture types of the distal limb, has not been previously described. A previous study describing intramodality and intermodality agreement between radiography and CT, highlighted the superior nature of CT for fracture diagnosis in distal limbs [5]. There are several studies describing the use of CT in a small cohort of single fracture types [3, 6–9]. There are also studies describing the use of intra-operative CT for a variety of fracture types [4, 10, 11] however, intra-operative CT is not widely available and poses a different set of benefits and challenges in comparison to pre-operative CT. A previous study described the use of pre-operative standing magnetic resonance imaging (MRI) for surgical planning of fracture repair [2]. This suggested that the use of pre-operative MRI significantly influenced surgical planning in most cases in the study and that standing pre-operative MRI was a safe and effective way to perform 3D imaging on a distal limb fracture [2].

The objective of this study is to describe the influence of immediate pre-operative CT on surgical planning in a variety of fracture types. Our hypothesis is that the use of immediate pre-operative CT will allow for more accurate surgical planning in comparison to radiography alone.

## Materials and methods

This study is a retrospective review of cases which presented to Rossdales Equine Hospital between 1^st^ January 2010 and 1^st^ December 2020 for further investigation and treatment of a suspected limb fracture. Cases were collated by the primary author via a clinical database search. The inclusion criteria were; cases presenting to the hospital with a suspected limb fracture where pre-operative radiographs were available for review and computed tomography (CT) was performed prior to further treatment. Cases were excluded where pre-operative radiographs were unavailable or appropriate lesion orientated projections had not been acquired. Case outcomes were not assessed in this study.

Due to the retrospective nature of this study, radiographic protocols for image acquisition could not be uniformly enforced. However, cases where standard radiographic views for visualisation of fracture configuration had not been acquired, did not meet the inclusion criteria for the study. The radiographic protocol and minimum standard included; four standard projections of the affected area (lateromedial, dorsopalmar/plantar, dorsolateral palmaromedial oblique, dorsomedial palmarolateral oblique), with additional lesion orientated oblique projections as deemed appropriate. The CT protocol is standardised for all fracture cases and includes acquisition of images inclusive of the affected bone or joint plus the proximal and distal articulations of the bone or bones affected. In cases of carpal or tarsal fractures the whole of the carpus/tarsus was imaged. All CT cases met this criteria.

**Fig 1:**
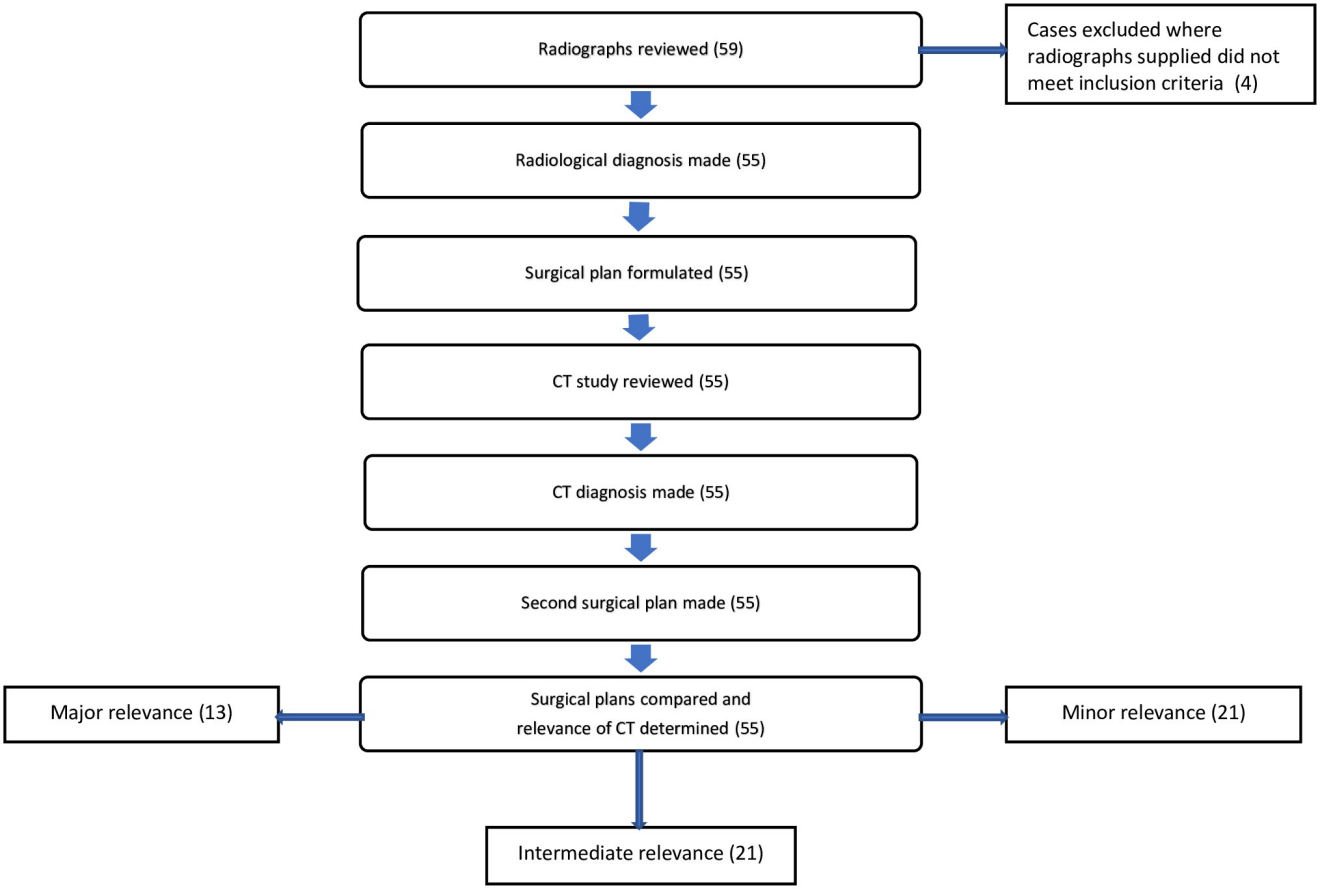
This diagram outlines the process by which decisions were made, relating to surgical planning and to the relevance of CT.

Each case was reviewed to determine the relevance of CT in the of surgical planning (Fig 1). The process was designed to simulate, as far as possible, the standard procedure for a patient admitted to the hospital with a limb fracture. The diagnostic images for each case were reviewed by a panel of three diplomates; two surgical (AB, MC) and one imaging specialist (VP). All decisions were made by consensus decision between the three members of the panel. Total time taken for decision making was not recorded in this study, however, decisions were made quickly, as if in real time. All parties were blinded to the diagnoses, surgical plans and outcomes of the cases that were made at the time of presentation, the panel were also blinded to any previous involvement they may have had in the case.

In each case, firstly, the radiographs were reviewed; and a radiological diagnosis agreed upon. A preliminary surgical plan was then formulated and recorded. The CT study was then reviewed, and again an imaging diagnosis was made. A second surgical plan was then formulated on the basis of CT images. The two surgical plans were then compared and finally each case was classified in to three categories on the basis of the relevance of the CT study to surgical planning. These groups were defined by previously published parameters by Genton et al, 2019 [2]. These are as follows;

**Major relevance**: defined as important new data was seen on CT images or surgical planning was significantly modified. For example, the screw axis/orientation was completely changed.
**Intermediate relevance**: defined as additional lesions were found on CT and/or surgical planning was slightly modified. For example, the screw placement was in the same axis but differing angle (i.e. the screw was in the same orientation but angled more dorsally).
**Minor relevance**: defined as CT studies confirmed the lesions and fracture configuration identified on radiography, without confirming the presence of additional complicating pathology that would alter the surgical plan. Whilst in these cases the CT did not provide additional information, it gave assurance of the fracture configuration and therefore remained beneficial surgical planning.

At the time of presentation to the hospital, all cases were subject to a clinical examination and radiography, unless adequate views had supplied from the referring veterinary surgeon. Once images had been acquired, they were reviewed and fracture configuration determined as far as was possible. CT was performed in all cases; most commonly due to fracture configuration being suspected to be more complex than was radiographically apparent. CT acquisition and fracture repair was performed under the same anaesthetic event in all cases. CT acquisition was performed using a multi-slice GE LightSpeed^®^ 16 slice helical scanner in a dedicated imaging suite. In most cases a single CT acquisition was performed. The field of view extended from one joint proximal and one joint distal to the fracture line. In some cases radio-opaque markers (skin staples) were placed and a second acquisition performed. Images were acquired, then reconstructed in both bone and soft tissue algorithms. In all cases, images were first reviewed by the operator for quality control, after which, each horse was rapidly transferred to the operating theatre via a wheeled padded surgical bed. CT images were then reviewed by the surgeon and an imaging specialist to establish fracture configuration and any additional pathology. A surgical plan was formulated and representative images were collated for reference in theatre. Prognosis for return to function was determined at this stage and discussed with connections of the horse. Some cases may have been subject to euthanasia where prognosis for return to function was poor, and depending upon the wishes of parties associated with the horse. In cases where surgical repair was a viable option, repair of the limb fracture was performed as per the formulated surgical plan. Monitoring of screw placement was by intra-operative radiography or fluoroscopy.

Fractures in this study were grouped into seven categories; first phalangeal fractures, second phalangeal fractures, carpal fractures, metacarpal, metatarsal fractures, tarsal fractures and sesamoid fractures. Within each group of fractures, each case was further categorised as far as possible using AO classification [12]. Anaesthetic time from the point of induction to the point of the horse being set up in the surgical suite following CT was recorded in all cases where the information was available. This information was taken from anaesthetic records, analysed as part of the clinical database search. Records where the exact timings were not clear were not included. Statistical analysis was performed using a simple Fisher’s exact test to compare proportions relating to significance of CT between the fracture groups, where P<0.05. P<0.05 was chosen as the level to represent a significant difference.

## Results

**Fig 2:**
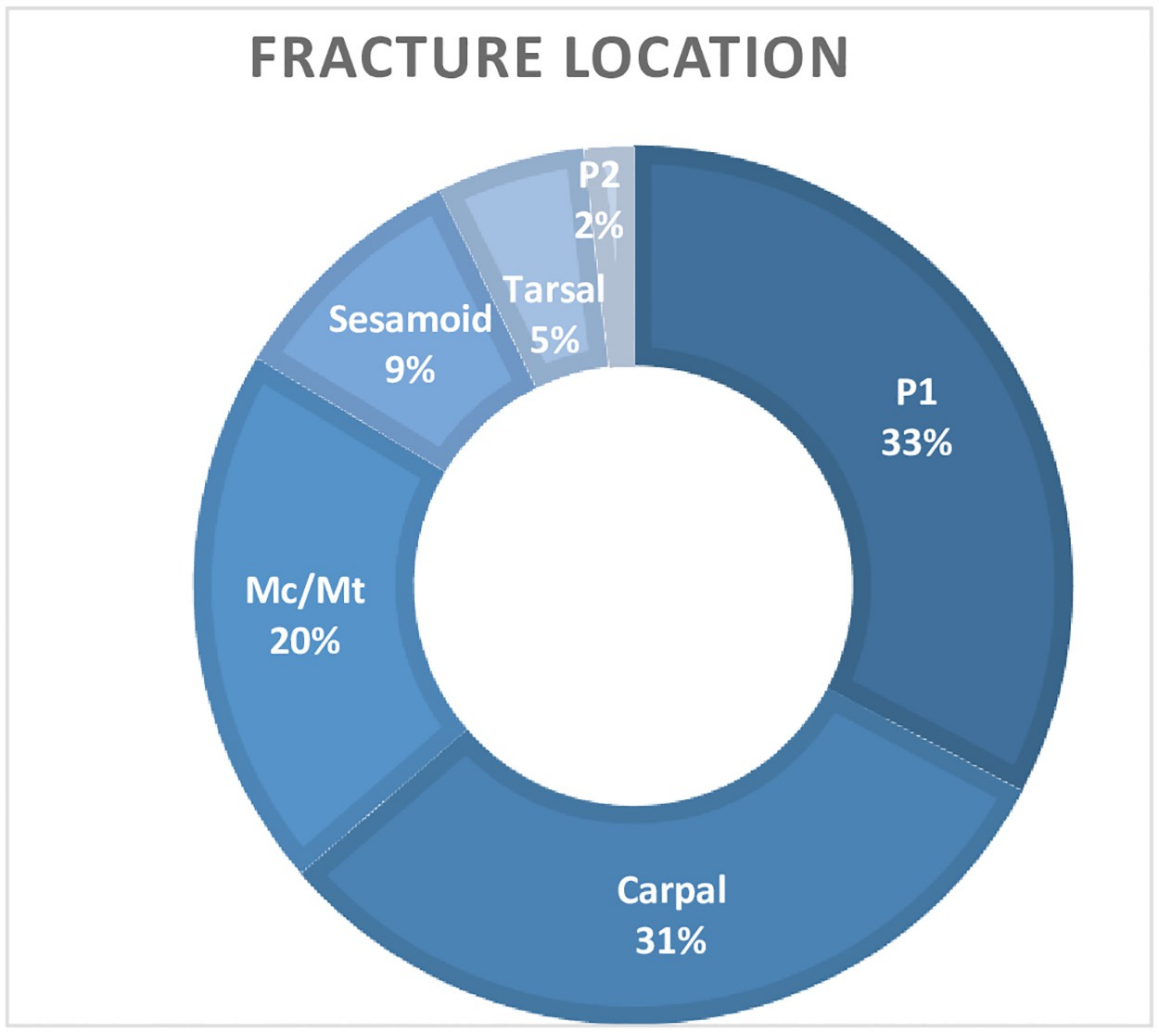
This chart details the distribution of fracture locations included in this study.

Fifty nine cases were collated following a clinical database search; 4 were excluded due to pre-operative radiographs not being available for review. The included cases were a variety of breeds including; 44 Thoroughbreds, 4 Warmbloods, 2 Arabians, 1 Polo pony, 1 Irish Draught, 1 Irish Sports Horse, 1 Welsh and 1 New Forest. The median age was 3 years (range 4 weeks-17 years). There were 26 females, 18 males, and 11 geldings. Of the 55 cases there were; 18 fractures of the proximal phalanx (33%), 17 fractures of the carpal bones(31%), 11 fractures of the metacarpal/tarsal bones (20%), 5 fractures of the proximal sesamoid bones (9%), 3 fractures of the tarsal bones (5%), and one fracture of the middle phalanx (2%) (Fig 2). Forelimb fractures were more common than hindlimb fractures in our study; 22/55 (40%, 95% CI [27%, 52%]) were of the right forelimb, 15/55 (27%, (95% CI [15%,39%]) fractures were of the left forelimb, 11/55 (20%, 95% CI [9%,30%]) were of the left hindlimb and 7/55 (13%, 95% CI [4%, 22%]) were of the right hindlimb. Anaesthetic time of the CT, from induction to arrival in theatre was available in 47/55 cases, mean anaesthetic time for CT was 26.7 minutes.

CT provided additional information or influenced a change in proposed surgical plan in the majority of cases in this study. In 13 of 55 cases (23.6%, 95% CI[12%,35%]) CT was of major relevance. In 21/55 cases (38.2%, 95% CI[25%,51%]) CT was of intermediate relevance. In 21/55 cases (38.2%, 95% CI[25%,51%]) CT was of minor relevance. Additional significant but unrelated findings were identified in 20/55 (36%, 95% CI[24%,49%]) cases, of which 9 were condylar fissure fractures.

### Proximal phalanx fractures

**Fig 3:**
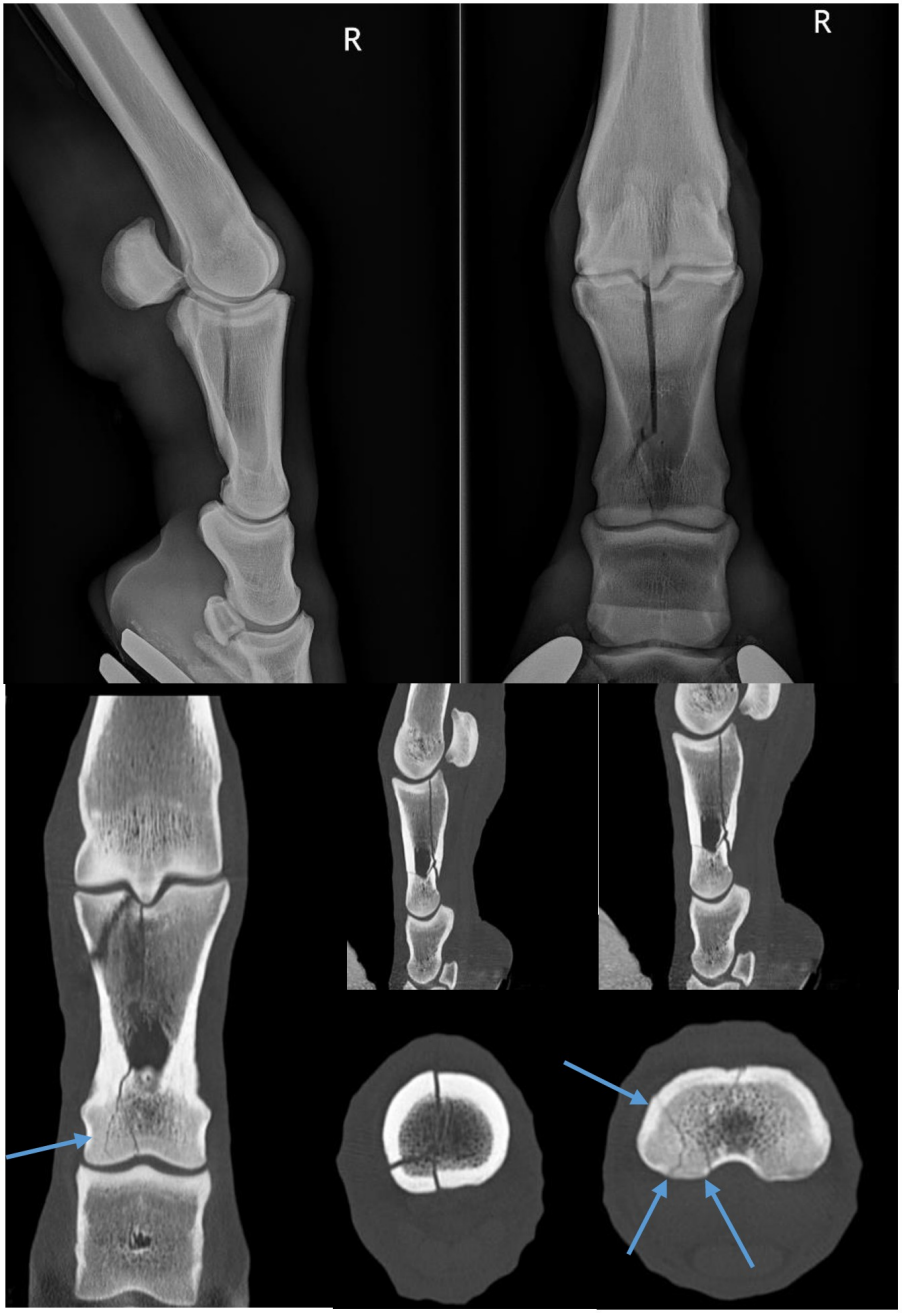
These are radiographic and CT images of a case in which CT was of major relevance to surgical planning. These images demonstrate radiographic evidence of a fracture of the proximal phalanx in sagittal and frontal planes and CT images confirm this but also highlight extensive distal fissure lines which were not radiographically appreciable. These additional findings significantly alter what is possible in terms of surgical repair.

Fracture configuration predominantly fell into a few distinct categories (Table 1). CT was defined as of major relevance in 3/18 cases (17%, 95% CI[0%, 34%]). One case, where CT was defined as being of major relevance, radiographically appeared to have a chronic frontal dorsoproximal P1 fracture, CT however confirmed the presence of an oblique frontal fracture and significant comminution, therefore significantly altering the surgical plan. The second case of major significance, radiographically appeared to have a complex configuration of fracture lines in frontal and sagittal planes. However, CT demonstrated only a simple parasagittal incomplete P1 fracture. This therefore simplified the surgical plan and changed the orientation of the screw placement. The final case had radiographic evidence of fracture lines in sagittal and frontal planes, CT confirmed these findings, but also identified multiple fissure lines at the distal extent of the fracture (Fig 3). CT was defined as of intermediate relevance in 8/18 cases (44%, 95% CI [22%, 67%]), and of minor relevance in 7/18 cases (39%, 95% CI [16%, 61%]). CT therefore changed the surgical plan or provided valuable additional information in the majority of proximal phalanx fractures (11/18, 61%, 95% CI [39%, 84%]).

**Table 1:** Proximal phalanx fractures.

Number	AO Classification	Further configuration
3	Complete uniarticular	All laterally exiting; two simple, one comminuted
10	Complete bi-articular	8 simple, one severely comminuted and displaced, one comminuted with articular fragments and proximal fissure lines
3	Short Incomplete	All sagittal; 2 acute, 1 chronic
1	Dorsoproximal eminence	Comminuted
1	Palmar eminence	Articular comminuted

Additional and unrelated pathology was also identified on CT in 7/18 cases (39%, 95% CI [16%, 61%]); there were 3 medial condylar fissure fractures, 2 lateral condylar fissure fractures, one case with a cyst like lesion of P2 and osteochondral fragment in the distal interphalangeal joint and one case with an apical medial sesamoid fracture and subchondral bone pathology of the medial metacarpal condyle. These additional findings had not been appreciated radiographically, although in 1/7 cases the additional finding would not have been within the radiographic field of view.

### Carpal bone fractures

**Fig 4:**
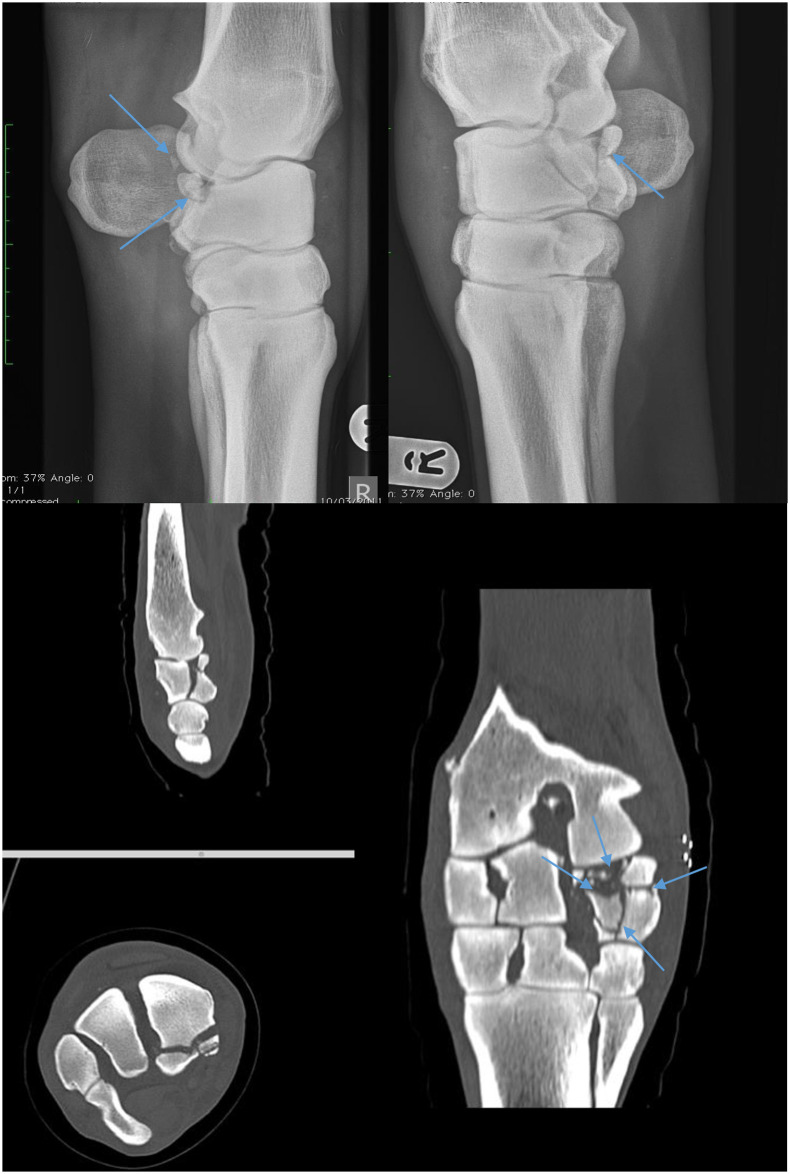
These are images of a carpal fracture where CT is of major significance. Following radiography the surgical plan had involved arthroscopic fragment removal, however following the CT diagnosis of severe comminution and partial joint collapse, the only viable surgical option was carpal arthrodesis. The severe comminution is labelled on this image.

There were 17 carpal bone fractures with a variety of configurations and locations (Table 2). CT was defined of being of major relevance in 7/17 cases (41%, 95% CI[18%, 65%]). One of which, radiographically, was diagnosed with a palmar intermediate carpal bone fracture and proximal radiocarpal bone fracture, however the CT diagnosis was a severely comminuted palmar radiocarpal bone slab fracture in the frontal plane, with numerous articular fragments and partial joint collapse, this therefore significantly changed the surgical plan from possible fragment removal to carpal arthrodesis (Fig 4). Another case had a radiological diagnosis of a third carpal bone slab fracture, however CT demonstrated no apparent fracture line only a prominent vascular channel, changing the treatment plan. CT was of intermediate relevance in 4/17 cases (24%, 95% CI[3%,44%]), and minor relevance in 6/17 cases (35%, 95% CI[13%,58%]). CT was therefore significantly relevant in the majority of carpal bone fractures (11/17, 65%, 95% CI[42%,87%]). In four cases additional unrelated findings were identified on CT; three horses had mild pathology within the proximal suspensory ligament, one horse had an avulsion fracture of the medial collateral ligament of the carpus and one horse had active lytic arthritic changes between the second and third metacarpal bones.

**Table 2:** Carpal bone fractures.

Number	AO Classification	Further Configuration
7	Third carpal slab	6 comminuted, 1 simple
2	Third and radiocarpal slab	Both comminuted C3 and simple radiocarpal fractures
1	Accessory carpal	Comminuted
1	Fourth carpal slab	Simple
1	Fourth, Intermediate and Ulnar Carpal	Comminuted fractures of C4, intermediate and ulnar carpal bones
1	Medial distal radial fracture, a radiocarpal bone avulsion fracture and fourth carpal bone slab fracture	
1	Radiocarpal slab	Comminuted
1	Intermediate carpal slab	Comminuted
1	Intermediate and radiocarpal slab	Both comminuted
1	No fracture–prominent vascular channel within third carpal bone	

### Metacarpal bone fractures

There were 6 metacarpal bone fractures; 5/6 were third metacarpal bone fractures and 1/6 was a fourth metacarpal bone fracture. Of the third metacarpal bone fractures; 3 were lateral condylar fractures and 2 were medial condylar fractures. Fracture configuration varied (Table 3). Of the lateral condylar fractures, CT was of major relevance in 2/3 (66.6%, 95% CI[10%,99%]) cases. One was severely comminuted with the appearance of a collapsed chronic palmar osteochondral lesion and the other was a long spiral fracture with dorsal and palmar comminution. CT was of intermediate relevance in 1/3 cases (33.3%, 95% CI[0%,87%]). Of the medial condylar fractures, CT was determined to be of minor significance in both cases. The CT of the fourth metacarpal bone fracture was of intermediate significance. CT of metacarpal fractures was therefore of significant relevance in the majority of cases (4/6, 67%, 95% CI[29%, 100%]). Additional findings were noted in one case; a medial condylar fissure was present in one case with a lateral condylar fracture.

**Table 3:** Metacarpal bone fractures.

Number	AO Classification	Further configuration
3	Lateral displaced	2 complete laterally exiting and comminuted
		1 comminuted, spiral
2	Medial spiral	1 spiral comminuted
		1 spiral simple
1	Fourth metacarpal	Oblique articular fracture with multiple avulsion fragments

### Metatarsal bone fractures

There were 5 metatarsal bone fractures; 4/5 medial condylar, and 1/5 lateral condylar (Table 4). In 3/5 (60%, 95% CI[17%, 100%]) cases CT was deemed to be of intermediate significance, and in 2/5 (40%, 95% CI[0%, 82%]) cases it was deemed to be of minor significance. Of the medial condylar fractures, CT was of intermediate relevance in 3 cases and minor relevance in one case. CT was deemed to be of minor relevance in the case of the lateral condylar fracture. Concurrent findings were identified in 2 cases; the case with a lateral condylar fracture had a medial condylar fissure fracture and one of the medial condylar fractures had a chronic lateral condylar fracture. CT was therefore significantly relevant to surgical planning in 3/5 cases (60%, 95% CI[17%,100%]).

**Table 4:** Metatarsal bone fractures.

Number	AO Classification	Further configuration
1	Lateral displaced	Complete, laterally exiting, with multiple articular fragments.
4	Medial spiral	All long, spiralling, 2 had articular fragments

### Proximal sesamoid bone fractures

There were 5 proximal sesamoid bone fractures, 4 medial and 1 lateral (Table 5). CT was of major relevance in the lateral sesamoid fracture. Of the four medial sesamoid fractures, CT was of intermediate relevance in 2/4 (50%, 95% CI[10%,99%]) cases and minor relevance in 2/4 (50%, 95% CI[10%,99%]) cases. CT was therefore of significant relevance in 3/5 cases (60%, 95% CI[17%,100%]). No additional unrelated pathology was identified in these cases.

**Table 5:** Proximal sesamoid fractures.

Number	Classification	Further configuration
4	Medial sesamoid	3 transverse mid-body of which one had a small articular fragment at the abaxial extent of the fracture line
		1 oblique transverse with associated articular fragments and suspensory ligament branch pathology
1	Lateral sesamoid	Complete, comminuted and basilar with fragments within the digital tendon sheath and superficial and deep digital flexor tendon pathology.

### Tarsal bone fractures

There were 3 tarsal bone fractures. One tarsal fracture was a comminuted articular lateral malleolus fracture, in this case CT was of intermediate relevance due to the CT confirmation of comminution and therefore change in surgical plan was made. In 2/3 (66.6%, 95% CI[13%,100%] cases CT was of minor relevance, both of these cases were central tarsal bone fractures; one was comminuted and displaced, the other was a simple complete sagittal slab fracture, but in both cases no further information was gained from CT, it only confirmed the original surgical plan. CT was therefore significant to surgical planning in 1/3 cases (30%, 95% CI[0%,87%]).

### Middle phalanx fractures

There was one middle phalanx fracture. The fracture configuration was complete bi-articular with a proximal articular fragment. In this case CT was of intermediate relevance, due to the identification of the articular fragment on CT.

### Statistical analysis

**Fig 5:**
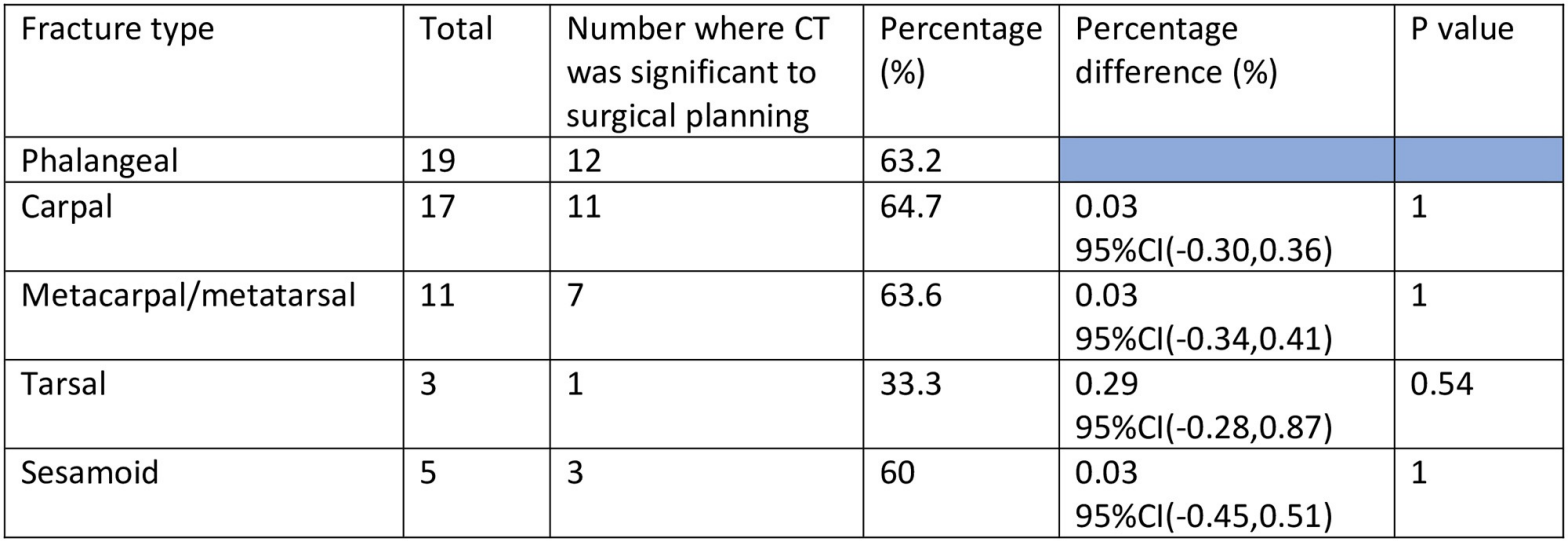
This figure demonstrates there to be no significant difference in relevance of CT on different fracture types where each group has been compared to the phalangeal fractures group.

Statistical analysis was subsequently performed. A Fisher’s exact test was used to assess any statistical difference in CT relevance between the groups of fracture types. Phalangeal fractures and metacarpal/metatarsal fractures were grouped together for the purpose of this, due to small sample sizes. This demonstrated no statistical difference in CT relevance between fracture types where p<0.05 (Fig 5). Tarsal fractures had a lower P value compared with the other groups but it was not statistically significant.

## Discussion

It is widely documented that in comparison to radiography alone CT offers greater spatial resolution and overcomes issues of superimposition to allow better visualisation of pathology in multiple planes [3]. It also allows visualisation of subtle changes in bone opacity compared with traditional 2D imaging [3]. This study found that the use of pre-operative CT for surgical planning of fracture repair provides essential additional information in the majority of cases with a limb fracture. In addition, this study suggests that immediate pre-operative CT is a safe and efficient option in place of intra-operative or standing pre-operative 3D imaging with no reported complications in this case series and an acceptable increase in anaesthetic time. In this study CT changed the surgical plan or provided relevant additional information about fracture configuration in 34 of 55 cases (62%). In 13 of these cases CT was of major relevance, suggesting the surgical plan based on the radiographic evidence alone would have been inappropriate, for example in our case series areas of comminution or fissure lines would have not been identified, potentially leading to propagation or further comminution of the fracture following fixation and almost certainly a poorer prognosis. In cases where CT was of intermediate relevance, again without the information gained from the CT study, the proposed surgical plan is likely to be suboptimal. In some of our cases CT highlighted increased complexity in fracture configuration where plate fixation was required instead of screws. In other cases, fracture configuration was more simple than expected, allowing for a more basic surgical plan and increasing the efficiency of the fixation, due to there being assurance of no further complicating pathology. In all 55 cases CT was deemed by the panel to provide greater confidence in the surgical plan. This area requires further research, with greater case numbers, but the increased surgical confidence seen in this study may in itself be enough to reduce surgical time and therefore optimise patient recovery in the long-term. These findings are supported by previous studies [3, 6] which focused on the use of CT in small cohort studies with a single fracture configuration or location.

### Timings

A well-documented concern with pre-operative CT is the additional anaesthetic time added to the surgical repair [3]. Data relating to total anaesthetic time attributable to CT was obtained from anaesthetic records in this study, data was available in 47/55 cases. The average time from induction of anaesthesia until the horse entered the surgical suite following CT acquisition was 26.7 minutes. This supports and exceeds the findings in previous studies which suggest that pre-operative CT should add no more than 30–45 minutes of anaesthetic time in a well-organised environment [3]. This therefore suggests that the use of pre-operative CT should not adversely impact the procedure or the anaesthetic risk profile in relation to timings. In our study, the specialist panel expressed that CT increased confidence in surgical planning in all cases, including those where additional information was not identified. This supports the previously documented information that additional information is likely to shorten the actual surgery time [3] due to increased surgeon confidence and more detailed and precise surgical planning. The use of pre-operative CT minimises the requirement for intra-operative imaging as implant placement has already been planned with CT so 2D imaging is only required for implant monitoring. This therefore significantly decreases the anaesthetic time attributable to further imaging, in comparison to cases undergoing 2D imaging only. It is also potentially more time efficient compared to the use of intra-operative CT due to the requirement to carefully drape and prepare the CT scanner for aseptic use during surgery.

### Procedure safety

Previous studies have reported complications such as further propagation or displacement of fractures [2] following induction of anaesthesia. This is mitigated against by providing external coaptation of the limb but complications can still occur. Not only does this worsen the prognosis for the patient, it also potentially, significantly changes the fracture configuration and therefore the surgical approach. Therefore in scenarios where pre-operative 3D imaging has been performed the imaging findings would no longer be accurate. This is a significant benefit of immediate pre-operative CT, where CT is performed after induction of anaesthesia so there is no risk of change of fracture configuration between CT and surgery. In this study there were no reported complications from induction of anaesthesia in relation to the fractured limb, however there was a risk that fracture pathology could have been exacerbated between radiography and CT. Measures were taken to prevent this including; external coaptation, controlled induction of anaesthesia with multiple people, and hoisting the horse excluding the fractured limb.

In this instance performing CT under the same anaesthetic event as surgical repair provides the most accurate and consistent information regarding fracture configuration compared with pre-anaesthetic imaging. In comparison to intra-operative CT, the approach taken in this study provides detailed information regarding fracture configuration, without the added complication of surgical preparation and draping around a CT machine, which is innately more complex than aseptically preparing an X-ray or fluoroscopy machine.

### Post-operative monitoring

Post-operative monitoring of fracture repairs is most commonly performed with radiography. Repeat CT examination would provide superior monitoring, due to requirement for general anaesthesia with our system, post-operative CT is not practical. This presents a benefit of intra-operative CT compared to pre-operative due to the ability for immediate post-operative CT review. However in a study of 86 cases of fracture repair using intra-operative CT only 4 cases received a post-operative CT [4] perhaps demonstrating an anecdotal lack of necessity for immediate post-operative CT.

### Study population

This study population represents a small proportion of the fracture repairs performed at this hospital, the more simple fractures are often repaired with the use of standing sedation and radiographic guidance without the use of CT.

### Additional findings

In 20 of the 55 cases there were significant additional findings, unrelated to the fracture being treated. These findings are deemed as significant because they may significantly alter the overall prognosis of the horse, due to the risk of another potentially catastrophic injury on returning to function. There were 9 metacarpal or metatarsal condylar fissure fractures identified. This may significantly alter the prognosis of these cases due to the propensity for further injury on return to function. These findings were identified on CT only. Whilst these findings are not directly related to this study, the relevance of these findings pose an interesting topic for research in the future.

### Limitations

This was a retrospective study which limits data to what can be found in clinical records and relies upon accurate record keeping.

It is likely that there is a level of inclusion bias in this study relating to the use of CT for fracture investigation. In this study, all cases which met the inclusion criteria and underwent a CT examination prior to fracture repair were included. The benefits and option of CT would have been discussed in most cases presenting with a fracture, where the clinician felt it may offer further information about fracture configuration. Clinically, CT will have been advised in those cases which radiographically had a complex fracture configuration, evidence of comminution or fissuring. Therefore, in some cases, CT may have been requested, despite it not being an essential diagnostic tool, due to certain economic or personal circumstances, including financial value of the horse, insurance status, athletic potential or personal value to the owner/trainer.

Further bias could have been introduced into this study due to the prior involvement of clinicians in included cases. This was minimised as far as possible by all members of the panel being blinded to the patient details, management, outcomes and clinician involvement in the cases. However two of the specialists had had some involvement in some of the cases in this study. This could potentially have influenced decision making in this study if they were able to identify the images and recall the cases. One member of the panel had no involvement in any case in this study, so would have no bias associated with their decision making.

This study was designed to, as far as possible, replicate a real time example of a horse presenting to the hospital for a CT and fracture repair. In reality, immediate pre-operative CT puts more time pressure on surgical decision making and image interpretation than, for example, pre-operative MRI the day prior to surgery. This is due to the horse being under general anaesthesia at the time of decision making, therefore review of images and formulation of surgical plans has to be as efficient as possible so as to not significantly increase anaesthetic time. This could lead to more subtle details of fracture configuration being missed and concurrent pathology perhaps not identified. This time pressure on decision making could not be fully captured in our study due to the retrospective review of images and consensus decision making, however an efficient and quick decision was made in each case and reviewers were not given extended periods of time to look at the images.

A limitation of immediate pre-operative CT in comparison with standing modalities is that in cases where the fracture configuration is more simple than expected, surgical repair under standing sedation may have been a viable option and therefore the horse would not have to undergo a general anaesthetic. However in this instance the horse is already under general anaesthesia at the time of examination and therefore the repair would also be performed under general anaesthesia. This also extends to cases in which no fracture was found, however of course without the CT, the horse may have undergone surgery instead.

Finally, statistical power in this study was low due to small numbers of cases. This could be improved in the future with a larger multi-centre study with higher case numbers.

## Conclusion

CT provided valuable information with regards to surgical planning in the majority of cases in this study. Performing the CT immediately pre-operatively can be beneficial and has minimal complications associated with it.

## Supporting information

S1 Data (XLSX)

## References

[1] Suarez Sanchez-Andrade J, Richter H, Kuhn K, Bischofberger AS, Kircher PR, Hoey S. Comparison between magnetic resonance imaging, computed tomography, and arthrography to identify artificially induced cartilage defects of the equine carpal joints. Veterinary Radiology and Ultrasound. 2018;59(3). doi: 10.1111/vru.12598 29455473

[2] Genton M, Vila T, Olive J, Rossignol F. Standing MRI for surgical planning of equine fracture repair. Veterinary Surgery. 2019;48(8). doi: 10.1111/vsu.13272 31270830

[3] Rose PL, Seeherman H, O’Callaghan M. Computed tomographic evaluation of comminuted middle phalangeal fractures in the horse. Veterinary Radiology and Ultrasound. 1997;38(6). doi: 10.1111/j.1740-8261.1997.tb00865.x 9402707

[4] Perrin RAR, Launois MT, Brogniez L, Clegg PD, Coomer RPC, Desbrosse FG, et al. The use of computed tomography to assist orthopaedic surgery in 86 horses (2002–2010). Equine Vet Educ. 2011;23(6).

[5] Crijns CP, Martens A, Bergman HJ, van der Veen H, Duchateau L, van Bree HJJ, et al. Intramodality and intermodality agreement in radiography and computed tomography of equine distal limb fractures. Equine Vet J. 2014;46(1). doi: 10.1111/evj.12082 23662918

[6] Kelmer G, Wilson DA, Essman SC. Computed tomography assisted repair of a central tarsal bone slab fracture in a horse. Equine Vet Educ. 2008;20(6).

[7] Braim AEP, Bell RJW, Textor JA, Lo WY, Puchalski SM, Galuppo LD. Computed tomography of proximal metatarsal and concurrent third tarsal bone fractures in a Thoroughbred racehorse. Equine Vet Educ. 2010;22(6).

[8] Gasiorowski JC, Richardson DW. Clinical use of computed tomography and surface markers to assist internal fixation within the equine hoof. Veterinary Surgery. 2015;44(2). doi: 10.1111/j.1532-950X.2014.12253.x 25132168

[9] Perez-Jimenez EE, Biedrzycki AH, Morton AJ, McCarrel TM. Three-dimensional printed guides for screw placement in equine navicular bones. Veterinary Surgery. 2021;50(4). doi: 10.1111/vsu.13616 33687084

[10] Andritzky J, Rossol M, Lischer C, Auer JA. Comparison of computer-assisted surgery with conventional technique for the treatment of axial distal phalanx fractures in horses: An in vitro study. Veterinary Surgery. 2005;34(2). doi: 10.1111/j.1532-950X.2005.00019.x 15860102

[11] Heer C, Fürst AE, del Chicca F, Jackson MA. Comparison of 3D-assisted surgery and conservative methods for treatment of type III fractures of the distal phalanx in horses. Equine Vet Educ. 2020;32(S10).

[12] Jörg Auer, Larry Bramlage, Patricia Hogan, Alan Ruggles, Jeffrey Watkins. AO Surgery Reference—Equine [Internet]. [cited 2022 Nov 16]. https://surgeryreference.aofoundation.org/vet/horse.

